# A novel intraperitoneal metastatic xenograft mouse model for survival outcome assessment of esophageal adenocarcinoma

**DOI:** 10.1371/journal.pone.0171824

**Published:** 2017-02-22

**Authors:** Md. Sazzad Hassan, Niranjan Awasthi, Jun Li, Margaret A. Schwarz, Roderich E. Schwarz, Urs von Holzen

**Affiliations:** 1 Department of Surgery, Indiana University School of Medicine, South Bend, IN, United States of America; 2 Harper Cancer Research Institute, South Bend, IN, United States of America; 3 Department of Applied and Computational Mathematics and Statistics, University of Notre Dame, Notre Dame, IN, United States of America; 4 Department of Pediatrics, Indiana University School of Medicine, South Bend, IN, United States of America; 5 Goshen Center for Cancer Care, Goshen, Goshen, IN, United States of America; 6 University of Basel, Basel, Switzerland; University of South Alabama Mitchell Cancer Institute, UNITED STATES

## Abstract

Esophageal adenocarcinoma (EAC) has become the dominant type of esophageal cancer in United States. The 5-year survival rate of EAC is below 20% and most patients present with locally advanced or widespread metastatic disease, where current treatment is largely ineffective. Therefore, new therapeutic approaches are urgently needed. Improvement of EAC patient outcome requires well-characterized animal models in which to evaluate novel therapeutics. In this study we aimed to establish a peritoneal dissemination xenograft mouse model of EAC that would support survival outcome analyses. To find the best candidate cell line from 7 human EAC cell lines of different origin named ESO26, OE33, ESO51, SK-GT-2, OE19, OACM5.1C and Flo-1 were injected intraperitoneally/subcutaneously into SCID mice. The peritoneal/xenograft tumor formation and mouse survival were compared among different groups. All cell lines injected subcutaneously formed tumors within 3 months at variable rates. All cell lines except OACM5.1C formed intraperitoneal tumors within 3 months at variable rates. Median animal survival with peritoneal dissemination was 108 days for ESO26 cells (5X10^6^), 65 days for OE33 cells (5X10^6^), 88 days for ESO51 cells (5X10^6^), 76 days for SK-GT-2 cells (5X10^6^), 55 days for OE19 cells (5X10^6^), 45 days for OE19 cells (10X10^6^) and 82 days for Flo-1 cells (5X10^6^). Interestingly, only in the OE19 model all mice (7/7 for 5X10^6^ and 5/5 for10X10^6^) developed bloody ascites with liver metastasis after intraperitoneal injection. The median survival time of these animals was the shortest (45 days for 10X10^6^ cells). In addition, median survival was significantly increased after paclitaxel treatment compared with the control group (57 days versus 45 days, p = 0.0034) along with a significant decrease of the relative subcutaneous tumor volume (p = 0.00011). Thus peritoneal dissemination mouse xenograft model for survival outcome assessment after intraperitoneal injection of OE19 cells will be very useful for the evaluation of cancer therapeutics.

## Introduction

Esophageal adenocarcinoma (EAC) has become the dominant type of esophageal cancer in United States. EAC now represents the fastest growing cancer in the western world. The incidence of EAC is increasing while the incidence of esophageal squamous cell remains unchanged [1–6]. Despite recent advances in surgical and radiation technique as well as in systemic medical treatment, prognosis of EAC remains poor [7–9]. The overall 5 year survival rate of EAC is below 20% and most patients present with locally advanced or widespread metastatic disease, where current treatment is largely ineffective [10, 11]. Therefore, new therapeutic approaches are urgently needed. Thus the poor survival rate of EAC patients warrants further evaluation of other anticancer drugs that block potential pathways of EAC progression.

Researchers often use a mouse model of esophageal cancer to evaluate these novel therapies prior to clinical protocol treatment [12–18]. Subcutaneous xenograft models are very commonly used for testing the efficacy of anticancer agents in many cancers including EAC. But mice subcutaneous EAC models only represent local tumor growth and do not provide any information about a survival benefit for a particular anticancer regimen, which is very crucial for experimental treatment efficacy. In addition, it has been observed that anticancer agents may well inhibit subcutaneous tumor growth without effecting overall animal survival [19]. One of the major obstacles in developing novel therapies for EAC has been the lack of an animal survival model for testing these anticancer pharmacotherapeutics. Thus improvement of esophageal adenocarcinoma patient outcome requires well-characterized animal survival models in which to evaluate novel therapeutics.

In this report, we present for the first time the successful establishment of a peritoneal dissemination mouse xenograft model for survival outcome analysis with intraperitoneal injection of human EAC cell lines.

## Materials and methods

### Ethics statement

All mouse experiments used in this study were carried out in accordance with the standards and guidelines of the Institutional Animal Care and Use Committee (IACUC) at the University of Notre Dame and confirmed to NIH guidelines. All animal researches used in this study were approved by the University of Notre Dame IACUC under protocol 15-08-263. At the end of experiments mice were euthanized by CO2 exposure followed by cervical dislocation according to University of Notre Dame IACUC-approved procedures.

### Cell lines culture and reagents

Human esophageal adenocarcinoma cell lines (ESO26, OE33, ESO51, SK-GT-2, OE19, OACM5.1C and Flo-1) were obtained from Sigma Aldrich (St. Lois, MO). All cell lines except Flo-1 were cultured in RPMI-1640 medium (Gibco, Grand Island, New York, USA) whereas Flo-1 was cultured in DMEM medium (Gibco) supplemented with 10% fetal bovine serum (Hyclone), 2 mM GlutaMax (Gibco), 100 U/ml penicillin, 100 mg/ml streptomycin at 37°C in a humidified atmosphere of 95% air– 5% CO_2_. ESO26, a loosely adherent cell line was established from a primary tumor located at the gastroesophageal junction and distal esophagus of a 56 year-old Caucasian male, OE33, an adherent cell line was established from the adenocarcinoma of the lower esophagus with Barrett’s metaplasia in a 73 year-old Caucasian female, ESO51, a suspension cell line was established from the distal esophagus with the presence of Barrett’s transformed mucosa in a 74 year-old Caucasian male, SK-GT-2, an adherent cell line was established from a poorly differentiated primary adenocarcinoma of gastric fundus in a 72 year-old Hispanic male, OE19, an adherent cell line was established from an adenocarcinoma of the gastric cardia/esophageal gastric junction in a 72 year-old Caucasian male, OACM5.1C, an adherent cell line was established from a lymph node metastasis derived from primary adenocarcinoma of the distal esophagus with the presence of Barrett’s transformed mucosa of a 47 year-old Caucasian female and Flo-1, an adherent cell line was established from a primary esophageal adenocarcinoma in a 68 year-old Caucasian male. Paclitaxel was bought from Hospira, Inc., Lake Forest IL 60045 and Carboplatin was bought from Sellersville, PA 18960.

### Peritoneal-disseminated animal survival model

Four to six week old female non-obese diabetic/severe combined immunedeficient (NOD/SCID) bought from Charles River were used in this study. All mice were maintained under pathogen free condition. Mice were intraperitoneally injected with 5X10^6^ to 10X10^6^ cells per mouse. Peritoneal tumor formation and animal survival were evaluated from the day of cancer cell injection until death. Animals were examined daily for signs of distress or development of jaundice and body weight was measured once a week. Animals were euthanized when they became moribund according to predefined criteria like rapid weight loss (>20%) or weight gain (>20% due to ascites), loss of ability to ambulate, labored respiration, or inability to drink or feed to avoid animal suffering [20, 21] in line with the local animal care committee protocol. After euthanasia animals were examined for the presence and extent of intraabdominal tumors. The peritoneal tumors and hepatic implants were harvested, immersion-fixed in 4% formaldehyde and paraffin-embedded. For microscopic examination, 5 μm thick tissue sections were obtained and stained with hematoxylin and eosin.

Mice survival studies were performed [22] with paclitaxel and carboplatin treatments. 10X10^6^ esophageal adenocarcinoma OE19 cells were intraperitoneally injected in each 4–6 weeks female SCID mouse. Mice were randomly grouped (n = 5 per group) two weeks after OE19 cells injection. Mice were treated intraperitoneally with vehicle, paclitaxel (20 mg/kg, 2 times a week for 2 weeks) or carboplatin (50 mg/kg, 2 times a week for 2 weeks). Animal survival was evaluated from the first day of treatment until death. Body weight was measured twice a week. Animals were euthanized when turning moribund according to above mentioned predefined criteria.

### Subcutaneous tumor growth model

Female NOD/SCID mice (4 to 6 weeks old) were subcutaneously injected with all seven esophageal adenocarcinoma cell lines (5X10^6^). Measurements of subcutaneous tumor size were started when mice had measurable tumors. The tumor size was measured twice a week for four weeks with with slide calipers and tumor volume (TV) was calculated as (W^2^XL)/2, where W is width and L is length of the tumor [23]. Relative tumor volume (RTV) was calculated according to the following formula; RTV = TV_n_/TV_0_ where TV_n_ is the tumor volume at the day of measurement and TV_0_ is the tumor volume on the first day of measurement [24].

Subsequent subcutaneous tumor growth study was performed where OE19 (5X10^6^) cells were subcutaneously injected in female NOD/SCID mice. All mice had measurable tumor two weeks after OE19 cell injection. The mice were then randomly grouped (n = 5 per group) and treated intraperitoneally as described earlier with vehicle, paclitaxel (20 mg/kg, 2 times a week for 2 weeks) [25] or carboplatin (50 mg/kg, 2 times a week for 2 weeks) [26]. Subcutaneous tumor size was measured twice a week for two weeks and TV with RTV was calculated as described earlier. Mice weight was measured twice a week during the period of the study. All mice were euthanized at the end of study.

### Cell viability assay

Cell viability of esophageal adenocarcinoma OE19 cell line was evaluated by the colorimetric WST-1 assay as previously described [27]. The measurement is based on the ability of viable cells to cleave the sulfonated tetrazolium salt WST-1 (4-[3-(4-iodophenyl)-2-(4-nitrophenyl)-2H-5-tetrazolio]-1,3-benzene disulfonate) by mitochondrial dehydrogenases. OE19 cell (4,000 cells/well) were plated in a 96-well plate in regular growth medium. After 16 hours the medium was replaced with 2% FBS containing medium and the cells were treated with paclitaxel or carboplatin (1 nM to 5 μM). After 72 hours, 10 μL WST-1 reagent was added in each well followed by additional incubation for 2 hours. The absorbance at 450 nm was measured using a microplate reader.

### Statistical analysis

The comparison of survival time between different groups was done by using the log-rank test, which is implemented in the "survdiff" function in the R [28] package "survival" [29, 30]. The comparison of the relative tumor volume (RTV) between treatment groups was done by first normalizing the RTV values at day 14 by the mean TRV value of the corresponding group at day 0, and then applying the two-sample t test, implemented in the "t.test" R function. p<0.05 was considered statistically significant.

## Results

### Establishment of a mouse model for peritoneal metastasis of EAC

To find the best candidate cell line for the establishment of a mouse model of peritoneal disseminated EAC we used seven EAC cell lines of various origin (Table 1).

**Table 1:** Cell lines used to establish peritoneal dissemination xenograft mouse model of esophageal adenocarcinoma.

Cell lines name	Cell lines origin
OE19	Adenocarcinoma of the gastroesophageal junction and gastric cardia (Caucasian)
OE33	Adenocarcinoma of the lower esophagus (Barrett’s metaplasia) (Caucasian)
ESO26	Adenocarcinoma of the gastroesophageal junction and distal esophagus (Caucasian)
Flo-1	Adenocarcinoma of the distal esophagus (Caucasian)
SK-GT-2	Adenocarcinoma of the gastric fundus (Hispanic male), poorly differentiated
ESO51	Adenocarcinoma of the distal esophagus (Caucasian) (Barrett’s metaplasia)
OACM5.1C	Adenocarcinoma of the distal esophagus (Caucasian)

All seven EAC cell lines except OACM5.1 C formed intraperitoneal tumors at variable rates (Table 2).

**Table 2:** Metastasis and survival outcome after intraperitoneal (IP) inoculation of esophageal adenocarcinoma cell lines.

Esophageal adenocarcinoma cell lines	Number of injected cells (IP)	Total number of mice used	Rate of colonization of peritoneum	Rate of bloody ascites	Median survival (Days)
OE19	5X10^6^	2+5 = 7	7/7	7/7	55
OE19	10X10^6^	5	5/5	5/5	45
OE33	5X10^6^	2	2/2	1/2	65
ESO26	5X10^6^	2	2/2	0/2	108
Flo-1	5X10^6^	2	2/2	1/2	82
SK-GT-2	5X10^6^	2	2/2	1/2	76
ESO51	5X10^6^	2	2/2	0/2	88
OACM5.1C	5X10^6^	2	0/2	0/2	>120

**Fig 1:**
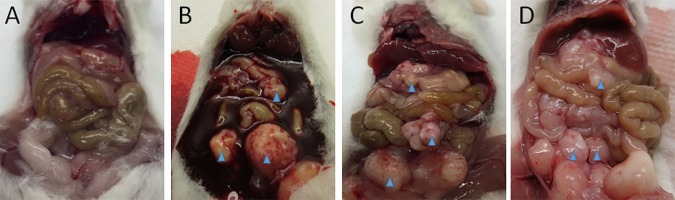
Formation of peritoneal tumor nodules and bloody ascites in SCID mice after intraperitoneal injection of 5X10^6^ cells. (A) No peritoneal tumor formation was observed even 4 months (120 days) after intraperitoneal injection of OACM5.1C cells. (B) Multiple peritoneal tumor formation with bloody ascites was observed ~55 days after intraperitoneal injection of OE19 cells. (C) Multiple peritoneal tumor formation but no bloody ascites was observed in OE33 cells ~65 days after intraperitoneal injection. (D) Similarly in SK-GT-2 cells multiple peritoneal tumor formation without bloody ascites was observed ~76 days after intraperitoneal injection. Blue arrows show the tumors.

**Fig 2:**
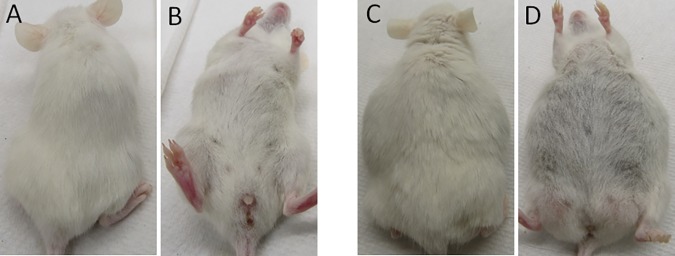
Ascites in SCID mice after intraperitoneal injection of 5X10^6^ cells. (A) & (B) There was no ascites observed in OACM5.1C cells. (C) & (D) Ascites was observed in OE19 cells.

**Fig 3:**
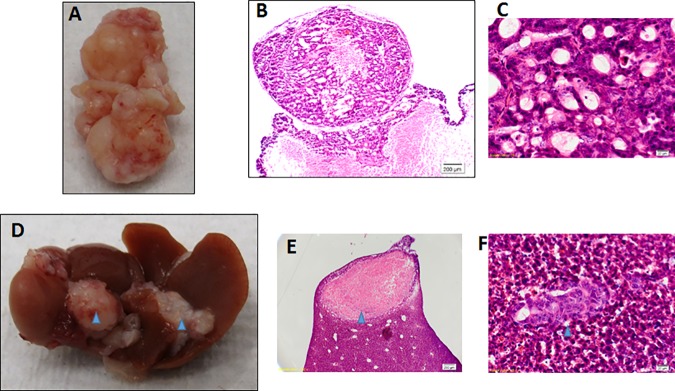
Peritoneal and hepatic tumor xenograft formation after intraperitoneal injection of OE19 cells. (A) Peritoneal tumor implant in SCID mice. (B) Low & (C) high power H&E stained sections of peritoneal tumors. (D) Hepatic tumor implants (blue arrows). (E) H&E staining of hepatic tumor implant (blue arrows). (F) H&E staining of OE19 metastasis to liver (blue arrows).

No peritoneal tumor was observed in mice injected with OACM5.1C even at 4 months (Figs 1A, 2A and 2B). After intraperitoneal injection of 5X10^6^ EAC cells in SCID mice earliest multiple peritoneal tumor formation was observed in the OE19 model, followed by the OE33 and the SK-GT-2 models (Fig 1B, 1C and 1D). Interestingly, only in the OE19 model all mice (7/7 for 5X10^6^ and 5/5 for10X10^6^) (Table 2) had bloody ascites (Figs 1B, 2C and 2D) with liver metastasis/implants (Fig 3) after intraperitoneal injection of cells. Bloody ascites within 2 months was observed in the OE19 (100% cases) and the OE33 (50% cases) models (Table 2). Especially with the OE19 injected mice, the ascites was almost entirely blood and there were very distinct cell aggregates within the ascetic fluid. Invasion of the omentum, the body wall and the diaphragm was also observed especially in the OE19 models.

### Animal survival in the peritoneal disseminated model

The longevity of the mice after intraperitoneal injection of EAC cell lines served as an indicator of the aggressiveness and tumorigenicity of the injected cells. Median survival time for each cell line is recorded in Table 2. Median animal survival with peritoneal dissemination was 108 days for ESO26 cells (5X10^6^), 65 days for OE33 cells (5X10^6^), 88 days for ESO51 cells (5X10^6^), 76 days for SK-GT-2 cells (5X10^6^), 55 days for OE19 cells (5X10^6^), 45 days for OE19 cells (10X10^6^) and 82 days for Flo-1 cells (5X10^6^). The most aggressive cell line OE19 resulted in a median survival time of less than 60 days. We therefore further characterized the animal survival in the OE19 model with more mice using variable numbers of OE19 cells (5X10^6^ and10X10^6^). We observed a very good animal survival time frame (45 days for 10X10^6^) for possible therapeutic interventions in an animal survival study.

### Effect of anticancer drugs on animal survival

**Fig 4:**
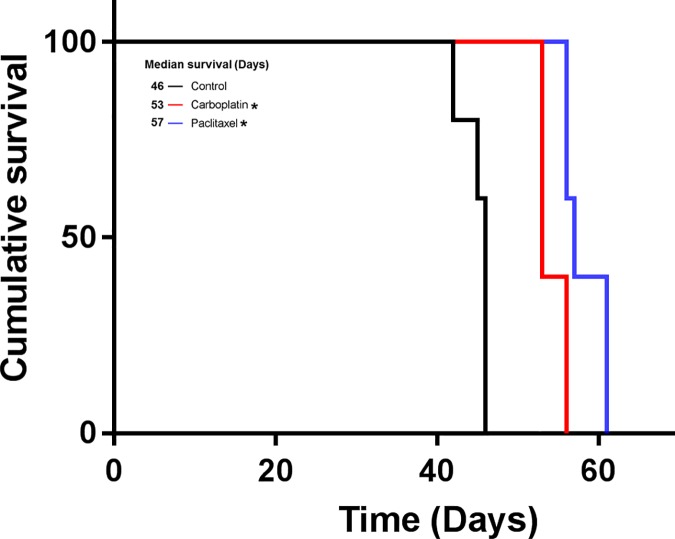
Mice survival in the OE19 peritoneal dissemination model treated with carboplatin and paclitaxel. 10X10^6^ OE19 cells were injected intraperitoneally in SCID mice and treatment started after 2 weeks and continued for another 2 weeks. The curve represents the animal survival time from the day of implantation. * Represents significant differences compared with control (vehicle) at p = 0.0034.

We chose the OE19 SCID mouse peritoneal disseminated model for the evaluation of therapeutic interventions. In this model, the animals were treated with anticancer drugs over a period of 14 days, starting 14 days after intraperitoneal injection of OE19 cells (10X10^6^). The median survival was 46 days in the control (vehicle) group. The median survival of mice was increased by paclitaxel treatment to 57 days (p = 0.0034, control versus paclitaxel) and also by carboplatin treatment to 53 days (p = 0.0034, control versus carboplatin); the p-value is exactly the same as control versus paclitaxel, since the log-rank test is a nonparametric test (Fig 4). The difference in survival between the two treatments are also statistically significant, with p = 0.018.

### Subcutaneous tumor growth and effect of anticancer drugs

**Fig 5:**
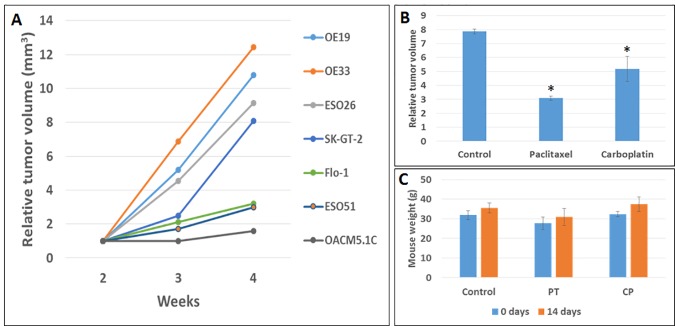
Study of subcutaneous tumor growth with changes of relative local tumor volume (RTV) and mouse weight after paclitaxel and carboplatin treatments in the OE19 subcutaneous mouse model. (A) Relative tumor volumes over a period of 4 weeks after subcutaneous injection of 5X10^6^ cells of seven esophageal adenocarcinoma cell lines. Enhanced relative tumor volumes were observed in OE33, OE19, ESO26 and SK-GT-2 cell lines. (B) RTV changes after paclitaxel and carboplatin treatments compared to control (vehicle) in OE19 subcutaneous mouse model. * indicates p<0.05 versus control. (C) No significant body weight change was observed after paclitaxel and carboplatin treatments compared to control in the OE19 subcutaneous mouse model.

All cell lines injected subcutaneously formed tumors within 3 months at variable rates (Fig 5A). Accelerated subcutaneous tumor growth was observed in the OE19 EAC cell line indicating its high tumorigenicity with aggressive phenotype in the animal model. Similar to the survival model, the OE19 subcutaneous tumor model showed therapeutic response to paclitaxel and carboplatin treatments (Fig 5B). The relative tumor volume (RTV) was decreased by 60.77% with paclitaxel (p = 0.00011) treatment and 34.34% with carboplatin (p = 0.0075) treatment. There was no significant decrease in animal weight in therapeutic groups (Fig 5C).

### Higher in vitro antiproliferative potency of paclitaxel over carboplatin on OE19 cells

We compared controls with two different treatments, paclitaxel and carboplatin, under different concentrations (1 nM, 10 nM, 50 nM, 250 nM, 1000 nM, and 5000 nM). We had four replicates in the control group, and in each concentration. First, we computed the proportion of cells surviving after each concentration, which was calculated by

$$proportion=1-\frac{measurement\;under\;a\;concentration}{mean\;measurement\;under\;control}.$$

**Fig 6:**
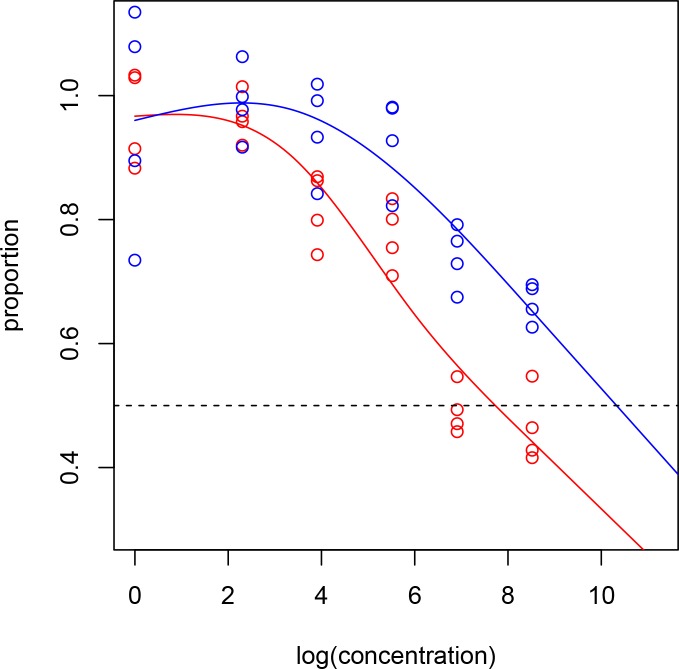
Higher antiproliferative potency of paclitaxel over carboplatin. OE19 cells were plated on 96-well plate and treated with 1 nM to 5000 nM concentrations of paclitaxel (red points) or carboplatin (blue points). After 72 hours, 10 μl WST-1 reagent was added in each well and incubated for 2 additional hours. The absorbance at 450 nm was measured using a microplate reader. The resulting number of viable cells was calculated by measuring absorbance of color produced in each well. Data are the mean ± SD of quadruplet

These proportions are shown as scatter plots in the Fig 6. The proportions from the paclitaxel treatment are shown as red points, and the proportions from the carboplatin treatment are shown as blue points. We log-transformed the concentrations to match our experimental design. It is clear that the relationship between proportions and log-concentrations are not linear. To reflect this, we fit a natural cubic spline [31] instead of a linear regression. The fitted splines are shown as red and blue lines for the two treatments. The log(IC50) values are 7.729 and 10.328, respectively. That is, the IC50 value is 2273 for the paclitaxel treatment, and 30577 for the carboplatin treatment. We saw that the IC50 value for the carboplatin treatment is about 13.45 times of the IC50 value for the paclitaxel treatment. To check whether this difference is statistically significant, we used the bootstrap to test the null hypothesis: IC50 of the two treatments are the same, versus the alternative hypothesis: IC50 of the carboplatin treatment is larger than the paclitaxel treatment. Based on ten thousand bootstrap resamplings, we get p-value = 0.0040, giving very strong evidence in rejecting the null hypothesis.

## Discussion

Establishment of subcutaneous and orthotopic esophageal adenocarcinoma (EAC) xenograft models has been reported previously [13, 18, 32–35]. However, there is no report of an EAC animal survival model after intraperitoneal injection of EAC cells. In this study, we established a novel animal model of peritoneal metastasis of EAC and evaluated the efficacy of most commonly used anticancer drugs for EAC therapy on animal survival. This study has important findings relevant to the development of new EAC survival models which will benefit EAC basic-research and preclinical drug-testing.

First, we succeeded in a reliable manner in establishing an EAC animal model of peritoneal metastasis in which peritoneal colonization of EAC cells occurred. To screen the best candidate cell line for this model, we used seven EAC cell lines in an attempt to establish a peritoneal dissemination model. After intraperitoneal injection of 5X10^6^ cells all cell lines except OACM5.1 C led to the development of peritoneal disseminated tumors in SCID mice at different time points. Among all these seven cell lines the earliest peritoneal tumor formation with shortest survival time was observed in the OE19 model. In addition, only in the OE19 model we persistently observed bloody ascites with liver metastasis. Regional lymph node metastasis has been observed by others after orthotropic injection of OE19 cells [32, 33]. Thus it seemed that the OE19 cell line was the best choice for a peritoneal metastatic model. We are therefore reporting the use of the OE19 cells for studying the survival outcome after peritoneal metastasis and consider it a useful model for examining new investigational therapeutic targets and agents for EAC. Our mouse survival model does not require any surgical procedure and is very simple to perform. Thus it is not only reliable and reproducible but also has a high degree of feasibility and is user friendly.

Secondly, we succeeded to observe a survival benefit in this OE19 model after treatment with clinically-proven anticancer drugs [36–38]. The main treatment for inoperable and metastatic EAC is systemic chemotherapy. Various anticancer drugs including paclitaxel and carboplatin have been used in recent years for treating patients with EAC [37]. However, severe side effects of using high dose or combination anticancer chemotherapeutics have limited their application. Therefore, new anticancer drugs including molecularly targeted agents must be developed. In our mouse survival model the median survival after intraperitoneal injection of 10X10^6^ OE19 cells was 46 days. This time frame is optimal for investigational therapeutic interventions with novel anticancer drugs with a sufficient duration to expect outcome differences. In our animal survival model, animal survival was significantly improved by paclitaxel and carboplatin injections, the standard anticancer agents frequently used clinically in EAC therapy.

Subcutaneous implantation xenograft and orthotropic models have been previously used for in-vivo experiments using EAC human cell lines for anticancer drug evaluation [14, 18, 32, 33, 39]. However, subcutaneous implantation models rarely metastasize and are not patient-like. In contrast, orthotropic models resemble human EAC disease progression more closely and frequently metastasize. Therefore they are considered to be the better option for studying EAC than the subcutaneous models. However, the establishment of an EAC orthotropic model is extremely difficult and is technically challenging to reproduce due to the anatomical location and small size of the mouse esophagus. In addition, it requires invasive procedures which can induce inflammation and thus may influence the efficacy of subsequent therapeutic interventions. Low invasiveness and cost effectiveness are some of the most important points for the ideal animal experiment. Accurate animal studies should be assessed with the least amount of outside influence possible. Thus, a simple, least invasive, patient-like EAC survival model with similar metastatic behavior has been needed.

Injection of cancer cells in the tail vein to implant cells in the lung and thus produce lung tumors is commonly used to study murine model of cancer lung metastasis [40, 41]. As EAC often metastasizes to the lung, tail vein injection can be employed to produce a survival model of EAC with lung metastasis. However, it has the disadvantages of formation of no lung tumors but instead only cancer cell colonization and of lacking the step of progression of the primary tumor to metastasis [40]. Our preliminary experiments with tail vein injection of OE19 cells also found cancer cell colonization with no lung tumor formation and failed to demonstrate a favorable time frame for survival outcome assessment. In addition, adenocarcinoma of the gastroesophageal junction commonly metastasizes to the peritoneal cavity and liver. Our OE19 survival model of diffuse peritoneal tumors with hepatic metastasis showed an optimal time frame to study the therapeutic response of metastasis. Thus, we have succeeded to produce patient-like tumor colonization of the peritoneal cavity and hepatic metastasis with the human EAC cell line OE19 by intraperitoneal injection in SCID mice. We used SCID mice over nude mice for the metastatic survival model because the survival effects were more reproducibly obtained in SCID mice than in nude mice. It took only 2 weeks at the earliest after intraperitoneal injection of OE19 to confirm metastatic nodules macroscopically. That is why we chose this time point to start treatment with our anticancer chemotherapeutics. Macroscopic metastatic nodules formation occurred for other EAC cell lines except OACM5.1C at later time points.

In summary, we were able to develop a reproducible, dependable and workable SCID mouse EAC peritoneal dissemination survival model with intraperitoneal injection of 10X10^6^ OE19 cells. Paclitaxel and carboplatin treatment showed efficacy both in local subcutaneous tumor growth and survival outcomes. This finding supports the novel and useful model for survival outcome analysis in EAC therapy research.

## Supporting information

S1 File In-vitro cell proliferation assay data. (XLSX)

S2 File Mice survival data. (PZFX)

S3 File Mice survival data. (XLSX)

S4 File Mice xenograft data. (XLSX)
