# Correction: A novel intraperitoneal metastatic xenograft mouse model for survival outcome assessment of esophageal adenocarcinoma

**DOI:** 10.1371/journal.pone.0180146

**Published:** 2017-06-19

**Authors:** The PLOS ONE Staff

The last author’s name is listed incorrectly in the citation. The correct citation is: Hassan MS, Awasthi N, Li J, Schwarz MA, Schwarz RE, von Holzen U (2017) A novel intraperitoneal metastatic xenograft mouse model for survival outcome assessment of esophageal adenocarcinoma. PLoS ONE 12(2): e0171824. https://doi.org/10.1371/journal.pone.0171824. The publisher apologizes for the error.
